# The time-dependent effects of bipolar radiofrequency energy on bovine articular cartilage

**DOI:** 10.1186/s13018-020-01626-5

**Published:** 2020-03-12

**Authors:** Liangquan Peng, Yusheng Li, Kai Zhang, Qi Chen, Lulu Xiao, Yiyun Geng, Yong Huang, Weimin Zhu, Wei Lu, Greg Zhang, Zhenhan Deng, Daping Wang

**Affiliations:** 1 Department of Sports Medicine, the First Affiliated Hospital of Shenzhen University, Shenzhen Second People’s Hospital, Shenzhen, 518035 Guangdong China; 2 Shenzhen Key Laboratory of Tissue Engineering, the First Affiliated Hospital of Shenzhen University, Shenzhen Second People’s Hospital, Shenzhen, 518035 Guangdong China; 3 Department of Orthopaedics, Xiangya Hospital, Central South University, Changsha, 410008 Hunan China; 4 Clinical Medical college of Shenzhen University, Shenzhen, 518000 Guangdong China; 5 Guangzhou Medical University, Guangzhou, 510182 Guangdong China; 6 Clinical College of Anhui Medical University, Affiliated Shenzhen Second Hospital, Shenzhen, 518035 Guangdong China; 7 McGovern Medical School, University of Texas Health Science Center at Houston, Houston, TX USA

**Keywords:** Radiofrequency energy, Chondroplasty, Articular cartilage, Chondrocyte, Scanning electron microscope

## Abstract

**Purpose:**

The purpose of this study was to compare the effect of bipolar radiofrequency energy (bRFE) on chondroplasty at the different time durations in an in vitro experiment that simulated an arthroscopic procedure.

**Methods:**

Six fresh bovine knees were used in our study. Six squares were marked on both the medical and lateral femoral condyles of each femur. Each square was respectively treated with bRFE for 0 s, 10 s, 20 s, 30 s, 40 s and 50 s. Full-thickness articular cartilage specimens were harvested from the treatment areas. Each specimen was divided into three distinct parts: one for hematoxylin/eosin staining histology, another for cartilage surface contouring assessment via scanning electron microscopy (SEM), and the last one for glycosaminoglycan (GAG) content measurement.

**Results:**

bRFE caused time-correlated damage to chondrocytes, and GAG content in the cartilage was negatively correlated to exposure time. bRFE caused time-correlated damage to chondrocytes. The GAG content in the cartilage negatively correlated with the exposure time. The sealing effect positively correlated with the exposure time. Additionally, it took at least 20 s of radiofrequency exposure to render a smooth cartilage surface and a score of 2 (normal) in the scoring system used.

**Conclusion:**

bRFE usage in chondroplasty could effectively trim and polish the cartilage lesion area; however, it induces a dose-dependent detrimental effect on chondrocytes and metabolic activity that negatively correlated with the treatment time. Therefore, cautions should be taken in the use of bRFE for treatment of articular cartilage injury.

## Introduction

Osteoarthritis results from the degeneration of articular cartilage and the ensuing secondary hyperostosis. Partial-thickness chondral defect has been found responsible for the degeneration of articular cartilage and remains without effective treatment methods [1, 2]. In recent years, bipolar radiofrequency energy (bRFE) ablation has gained popularity among arthroscopic knee surgeries as a solution to articular cartilage lesions; it creates a smooth, stable cartilage surface by thermal shrinkage or removal of fibrillated cartilage and thus stops the progression of chondral degeneration [3].

After studying chondromalacic human cartilage, Edwards et al. [4] reported that radiofrequency caused cellular death at 2 mm depth, and the damage even reached subchondral bone among 13 out of 20 specimens. In an in vitro arthroscopic experiment, Ryan et al. [5] highlighted that bRFE hindered cartilage vitality and damaged its matrix when the power was above 20 W. Therefore, when bRFE is applied to chondral conditions, the power should be controlled within 20 W, and the safety profile of radiofrequency remains yet to be proved in long-term studies. Voloshin et al. [6] demonstrated chondroplasty with bRFE was indeed effective for partial-thickness chondral defects, but it is still awaiting long-term results. A recent systematic review showed that bRFE ablation achieves dramatically better clinical outcomes and lower complication rates in the treatment of chondral defects than a mechanical shaving device [7]. However, thermal chondroplasty using bRFE ablation also arouses concerns about the risk of osteonecrosis, chondrolysis, and progression of partial-thickness chondral lesions [8].

In summation, the safety and long-term effects of bRFE, especially in regards to chondral lesions, are major concerns of practitioners and patients [9]. Therefore, the current study aimed to investigate how the different exposure times of bRFE affect the vitality of chondrocytes and cartilage surface. The study simulated the clinical treatment of arthroscopic procedure in an in vitro setup and applied a constant level of power to the radiofrequency. Our study will contribute to current literature on the clinical use of radiofrequency in resolving chondral defects.

## Materials and methods

### Subjects

The animal experiment was carried out in accordance with relevant guidelines and regulations and was approved by the Medical Ethics Committee of the First Affiliated Hospital of Shenzhen University. Fresh adult bovine knees purchased from the Guangdong Medical Laboratory Animal Center were used in this experiment. The animals were around 18 months old with closed epiphyses. The entire knee joint was harvested with the joint capsule intact and stored at 4 °C and delivered to the lab within 1 day. Chondrocyte viability was maintained during a 3- to 6-day period of storage, which has been demonstrated by a previous publication [10]. The experiment was performed 1 day after the animals’ death.

### Experimental model

**Fig. 1:**
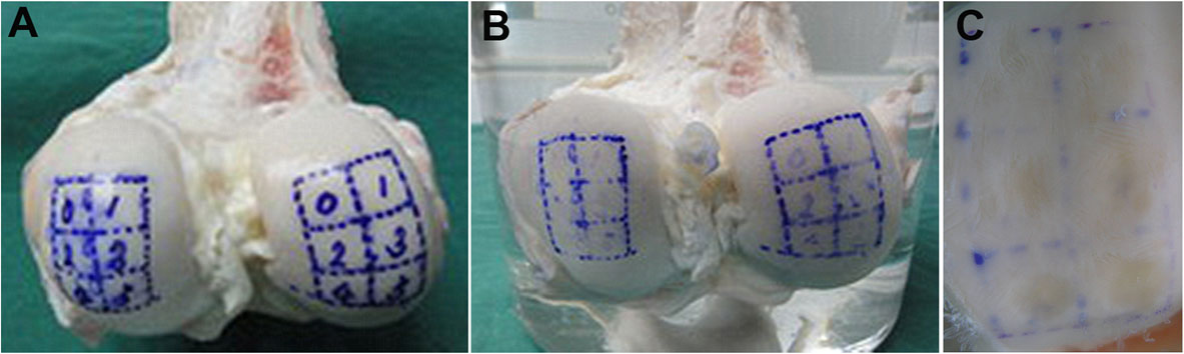
**a** Groups at the medial and lateral distal bovine femoral condyles. The 0-s group, treated with bRFE for 0 s; the 10-s group, treated with bRFE for 10s; the 20-s group, treated with bRFE for 20 s; the 30-s group, treated with bRFE for 30 s; the 40-s group, treated with bRFE for 40 s; the 50-s group, treated with bRFE for 50 s. **b** Medial and lateral distal bovine femoral condyles before RFE treatment. **c** Medial and lateral distal bovine femoral condyles after RFE treatment

Under sterile conditions, a model of grade-II cartilage degeneration was established by opening the knee joints, exposing the articular cartilage on the femoral condyles, scraping chondral membrane off with scalpel, and abrading the cartilage on both the medial and lateral condyles with a rasp [5]. Grids consisting of six squares of 1 × 1 cm^2^ area were created on the medial and lateral femoral condyles at the weight-bearing area with a sterile ink marker. Each grid was marked with a number from 0 to 5 (Fig. 1a). Zero represented the control without any bRFE treatment, while the others served as the experiment groups treated with different time durations of bRFE (*N* = 6/group).

### Treatment with bRFE

Radiofrequency ablation was generated from the SAPHYRE 60° angle bipolar ablation probe on the Vulcan EAS electrothermal system (Smith and Nephew, Inc., Andover, MA, USA). The probe was set at bipolar ablation mode at 70 W. The probe was moved in a meandering pattern in contact with the cartilage surface but not applying and pressure; in addition, no fluid flow was used during bRFE treatment. The model was submerged in normal saline in order to simulate the arthroscopic environment. Squares no. 1 to 5 were treated in the aforementioned method for 10, 20, 30, 40, and 50 s, respectively. After the treatment, the squares were taken off one by one with a scalpel blade ensuring the removal of full-thickness cartilage containing subchondral bone. Each cartilage square was dissected into three parts: part A, B, and C. Part A was used for hematoxylin/eosin (HE) staining. Part B was used for observation of the cartilage contour under scanning electron microscope (SEM). Part C was used for the measurement of glycosaminoglycan (GAG) content in the articular cartilage.

### Histology

All the part A’s from the six groups were fixed in 10% neutral buffered formalin (NBF) for 2 days. Then, they were decalcified and paraffin-embedded. Sections were cut at 5-μm thickness using a microtome, deparaffinized through xylene, and hydrated via ethanol gradient and water. Afterwards, HE staining was performed to reveal the cartilage morphology. The slices were dyed in hematoxylin solution for 5 min, given a 1-min water soak, differentiated with 1% hydrochloric acid ethanol for 30 s, given a 15-min water soak, dyed with 0.5% eosin for 3 min, given a distilled water soak, and finally sealed for observation after dehydration.

### SEM observation

Part B was fixed in 10% NBF, dehydrated in a graded series of ethanol, dried at critical point, and coated with gold in an Autoconductavac IV (Seevac, Pittsburgh, PA) before their contouring was examined with SEM (Hitachi S 3000 N, Tokyo, Japan) and scored according to the system provided in Table 1 [11]. A higher score indicates a smoother articular cartilage surface.

**Table 1:** SEM grading system for chondromalacic cartilage surface after bRFE treatment

Surface of chondromalacic cartilage	Score
Extremely smooth	3
Relatively smooth with melted fronds	2
Rough and irregular with melted fronds	1
Rough and irregular with fronds	0

If a surface was graded to be between 2 scores, the mean of the 2 scores was used (0.5, 1.5, 2.5)

### GAG content

GAG content was measured using the described dimethylmethylene blue (DMB) method [12]. After freeze-drying for 1 day, the cartilage specimens (part C) were weighed on an electronic scale to determine their dry weight. Then, they were immersed in papain at 60 °C 1 day for enzymolysis. Three milliliters of DMB was added for every 100 μl of the solution. The optical density (OD) value was determined using a UV spectrophotometer. Compared with standard curves, the GAG content of each specimen was calculated using the following formula: GAG content (μg/mg) = GAG content of the specimen/dry weight of the specimen.

### Statistical analysis

Sample results are presented in the text as mean ± standard deviation. The software SPSS 16.0 (version 15.0 for Windows; SPSS Inc., Chicago, IL, USA) was applied for statistical analysis and management. The one-way analysis of variance (ANOVA), SNK-q, and Dunnett’s T3 were applied for comparisons of multi-sample means and heterogeneity of variance. Results were considered significant at a value of *P* < 0.05.

## Results

### Macroscopic observation

The gross appearance of the articular cartilage before and after rRFE treatment was presented in Fig. 1b, c. The control group showed a rough and uneven cartilage surface with a bright white color. In the 10-s and 20-s treatment groups, the cartilage fragment was dissolved, and the surface was slightly rough without any change in color. In the 30-s and 40-s treatment groups, the surface was smoother with a light yellow color. In the 50-s treatment group, the surface was smooth and became yellow in color.

### Histology

**Fig. 2:**
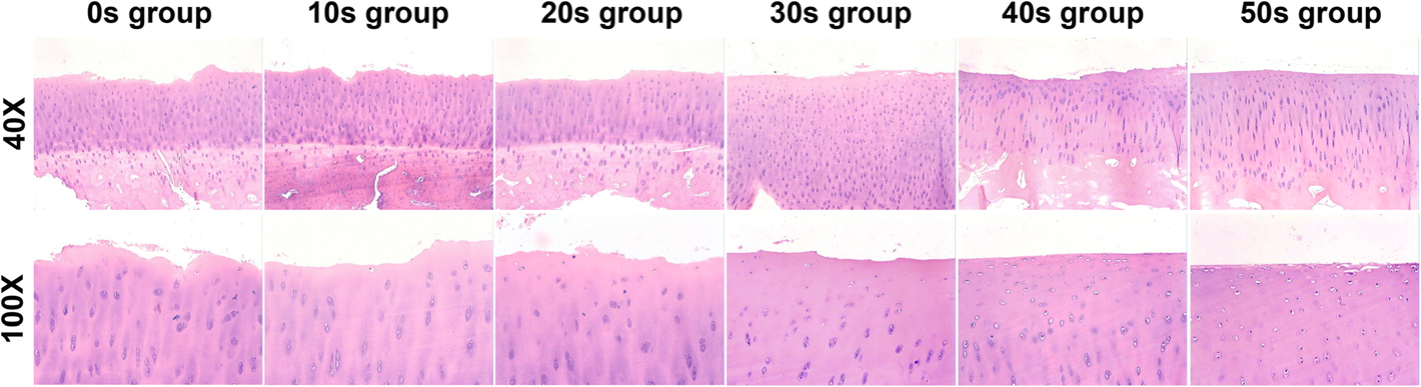
HE staining showed the cartilage morphology in each group after different duration times of bRFE treatment

We observed cracks on the rough surface and chondrocytes with normal morphology orderly arranged in each layer in the control group. The cartilage surface became smoother, and the chondrocytes appeared more vacuolated, had a disorderly arrangement, and decreased in number along with the increasing bRFE treatment time in the experiment groups (Fig. 2).

### SEM examination

**Fig. 3:**
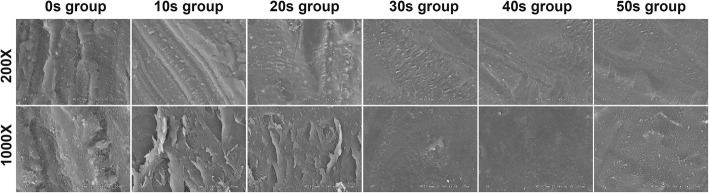
SEM examination of the cartilage morphology in each group after different duration times of bRFE treatment

**Fig. 4:**
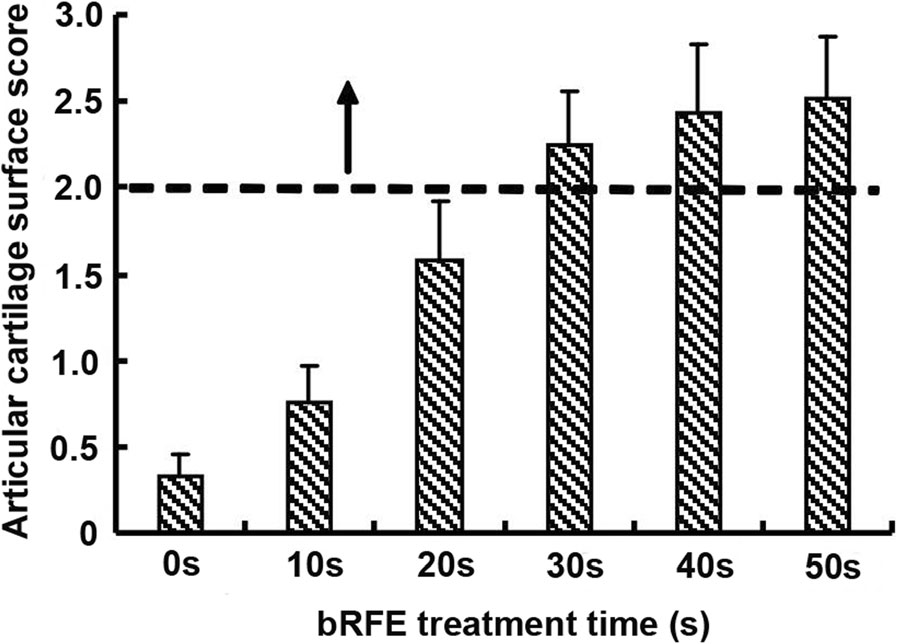
Score of cartilage surface in each group after bRFE treatment at different duration times via SEM examination. The dotted line at surface score 2 is a standard for cartilage surface smoothing

The results of SEM examination indicated a positive correlation between smoothness of cartilage surface and the exposure time to bipolar frequency (Fig. 3). The cartilage surface did not become smooth and the score did not reach 2 (normal) until 20 s of frequency exposure. The SEM score was remarkably higher among 20-s, 30-s, 40-s, and 50-s treatment groups than the 10-s treatment and control groups. Comparison of SEM scores among groups revealed that a statistically significant difference was present in all the results except for that between the 30-s and 40-s groups as well as that between the 40-s and 50-s groups (Fig. 4).

### GAG content

**Fig. 5:**
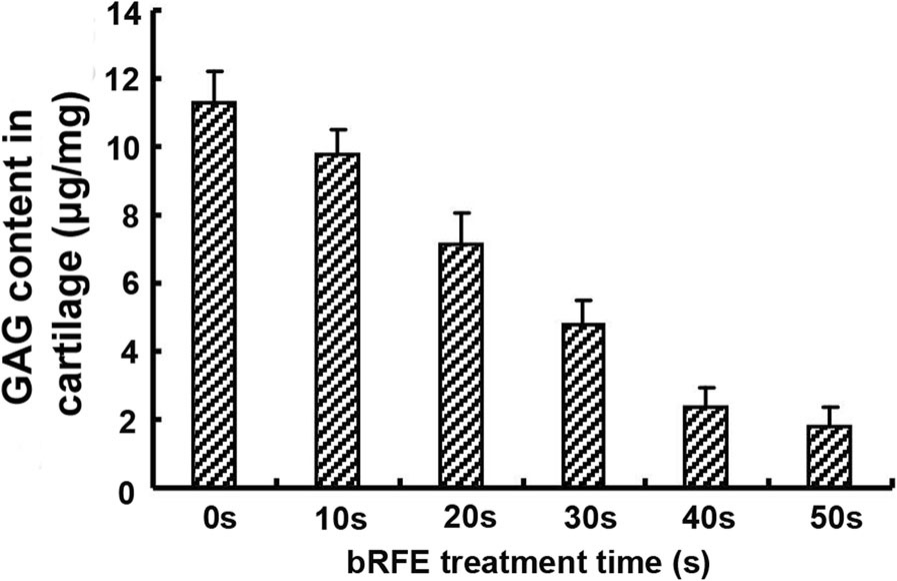
GAG content in the cartilage in each group after bRFE treatment at different duration times

The GAG content in the cartilage specimens was negatively correlated with the bRFE exposure time. All of the pair-wise comparisons between groups were statistically significant except for that between the 40-s and 50-s treatment groups (Fig. 5).

## Discussion

Chondroplasty is commonly applied to chondral lesions in clinical practice, although there are multiple alternatives. Laser is the first approach to chondroplasty with valid efficacy, but was later rendered obsolete due to iatrogenic subchondral osteonecrosis and its high cost [13, 14]. On the other hand, mechanical debridement (with shaver) intends to eliminate fibrillated or loose cartilage; however, it also removes healthy surrounding articular cartilage and does not improve the clefts and instability on the cartilaginous surface [15, 16].

Recently, thermal chondroplasty with radiofrequency has gained a wider acceptance. Monopolar and bipolar radiofrequency devices still prevail in clinical practice. The outcome of radiofrequency is affected by multiple variables, including treatment protocol, power setting, exposure time, and the temperature and speed of fluid flow. As safety and efficacy are the prerequisites for radiofrequency in chondroplasty [17], our study reproduced the actual surgical model to facilitate the observation of the impact of exposure time of bRFE on chondrocyte vitality and surface contouring under simulated arthroscopic environment and constant power delivery. These two major outcome measures enabled us to evaluate the safety and efficacy of this treatment method.

A previous study had demonstrated that the heat produced by bRFE could damage both chondrocytes and matrix [18]. GAG, produced and released by chondrocytes and a major ingredient of chondroid matrix, fills in the network built by type-II collagen. Kaplan et al. [19] revealed that radiofrequency sealed the cartilaginous surface while sparing the surrounding chondrocyte and matrix. Viable cell staining followed by confocal laser microscopy has been commonly used in research into bRFE-induced chondrocytic death, and signs of compromised vitality and metabolic activity of chondrocytes adjacent to the treatment zone have been noticed [10]. Hence, the GAG content could better reflect the physiological health of chondrocytes. This perspective is underpinned by the fact that proteoglycan has a significantly higher metabolic rate than collagen, making it feasible to use on an in vitro assessment of chondrocyte vitality [5, 18]. It has been demonstrated that the volume of GAG released from cartilage dropped as exposure time increased under constant energy output, indicating that prolonged exposure results in lower cartilage metabolic activity.

Cartilage smoothness may be overestimated under arthroscopic exploration due to its lower magnification power than SEM, leading to insufficient sealing of the clefts at the end of the procedure [11]. In our study, the score of 2, indicating a relatively smooth contour according to the SEM grading system, was only possible when the cartilage was exposed to bRFE for longer than 20 s. The cartilage smoothness being correlated to the exposure time of bRFE is consistent with that of Lu et al. [11].

Bovine knee was selected as the specimen based on the similarity in thickness to human articular cartilage as well as shared biomechanical and biochemical profiles [20]. No fluid flow was used in this study, as Edwards et al. [21] revealed a significant increase in temperature at 500 μm underneath the cartilage surface but observed no change of temperature at the depth of 200 μm and 2000 μm when a 120-ml/min flow rate was applied in bRFE.

This study also presents with some limitations. Firstly, there were differences between the artificially induced chondral degeneration and the naturally and slowly occurring one. Secondly, the experiment was in vitro and unable to simulate ideal in vivo conditions. Thirdly, no fluid flow was applied while the actual arthroscopic procedure performed on patients requires fluid flow of varying speeds. Hence, caution is required when one tries to translate our conclusions into clinical settings, as the conclusions of a particular bRFE device come only from that device under the adopted treatment protocol. A different set of outcomes can be expected when any of the variables are changed (e.g., the probe is moved over the cartilage surface in a non-contact manner).

In conclusion, bRFE usage for chondroplasty could effectively trim and polish the cartilage lesion area. However, it induced a dose-dependent detrimental effect on chondrocytes and metabolic activity that negatively correlated with the treatment time. Therefore, cautions should be taken in the use of bRFE for treatment of articular cartilage injury.

## Data Availability

All data generated or analyzed during this study are included in this published article.

## Abbreviations

ANOVA: One-way analysis of variance
bRFE: Bipolar radiofrequency energy
DMB: Dimethylmethylene blue
GAG: Glycosaminoglycan
HE: Hematoxylin/eosin
NBF: Neutral buffered formalin
OD: Optical density
SEM: Scanning electron microscopy
